# Phenotyping seedlings for selection of root system architecture in alfalfa (*Medicago sativa* L.)

**DOI:** 10.1186/s13007-021-00825-3

**Published:** 2021-12-07

**Authors:** Bruna Bucciarelli, Zhanyou Xu, Samadangla Ao, Yuanyuan Cao, Maria J. Monteros, Christopher N. Topp, Deborah A. Samac

**Affiliations:** 1 Department of Agronomy and Plant Genetics, University of Minnesota, 1991 Upper Buford Circle, St. Paul, MN 55108 USA; 2 USDA-ARS, Plant Science Research Unit, 1991 Upper Buford Circle, St. Paul, MN 55108 USA; 3 Department of Plant Pathology, University of Minnesota, 1991 Upper Buford Circle, 495 Borlaug Hall, St. Paul, MN 55108 USA; 4 Noble Research Institute, 2510 Sam Noble Parkway, Ardmore, OK 73401 USA; 5 Donald Danforth Plant Science Center, 975 N Warson Road, Olivette, MO 63132 USA; 6 Present Address: Kohima Science College, Jotsoma, 797002 Nagaland India; 7 Present Address: School of Life Sciences, Anhui Agricultural University, Hefei, 230036 Anhui China; 8 Present Address: Bayer Crop Science, Chesterfield, MO 63017 USA

**Keywords:** Alfalfa, Branch root, Root system architecture, Seedling phenotyping, Tap root

## Abstract

**Background:**

The root system architecture (RSA) of alfalfa (*Medicago sativa* L.) affects biomass production by influencing water and nutrient uptake, including nitrogen fixation. Further, roots are important for storing carbohydrates that are needed for regrowth in spring and after each harvest. Previous selection for a greater number of branched and fibrous roots significantly increased alfalfa biomass yield. However, phenotyping root systems of mature alfalfa plant is labor-intensive, time-consuming, and subject to environmental variability and human error. High-throughput and detailed phenotyping methods are needed to accelerate the development of alfalfa germplasm with distinct RSAs adapted to specific environmental conditions and for enhancing productivity in elite germplasm. In this study methods were developed for phenotyping 14-day-old alfalfa seedlings to identify measurable root traits that are highly heritable and can differentiate plants with either a branched or a tap rooted phenotype. Plants were grown in a soil-free mixture under controlled conditions, then the root systems were imaged with a flatbed scanner and measured using WinRhizo software.

**Results:**

The branched root plants had a significantly greater number of tertiary roots and significantly longer tertiary roots relative to the tap rooted plants. Additionally, the branch rooted population had significantly more secondary roots > 2.5 cm relative to the tap rooted population. These two parameters distinguishing phenotypes were confirmed using two machine learning algorithms, Random Forest and Gradient Boosting Machines. Plants selected as seedlings for the branch rooted or tap rooted phenotypes were used in crossing blocks that resulted in a genetic gain of 10%, consistent with the previous selection strategy that utilized manual root scoring to phenotype 22-week-old-plants. Heritability analysis of various root architecture parameters from selected seedlings showed tertiary root length and number are highly heritable with values of 0.74 and 0.79, respectively.

**Conclusions:**

The results show that seedling root phenotyping is a reliable tool that can be used for alfalfa germplasm selection and breeding. Phenotypic selection of RSA in seedlings reduced time for selection by 20 weeks, significantly accelerating the breeding cycle.

## Introduction

Alfalfa (*Medicago sativa* L.) is the most widely cultivated forage legume worldwide and the third most widely produced crop in the United States, with an annual direct value of $10.8 billion in 2019 [1]. Alfalfa production is essential for sustaining the dairy and beef industries, which used an estimated 53 million tons of alfalfa and alfalfa mixtures harvested from approximately 6.9 million hectares in the United States in 2018 [2]. As a perennial crop, alfalfa provides environmental benefits and contributes nitrogen to subsequent crops in rotations [3, 4]. Alfalfa also has attributes suitable for use as a bioenergy feedstock [5, 6]. However, alfalfa acres in farming systems have declined steadily due in part to the stagnation in biomass yields [7–9].

In alfalfa and other crops, selection for biomass accumulation or grain yield has focused primarily on the above ground plant traits, largely ignoring the contribution of the root system to improve nutrient acquisition and yields [10–14]. Root system architecture (RSA) encompasses the spatial and temporal organization of roots in the growth medium, and thus greatly influences the water and nutrient capturing abilities of a plant. In alfalfa, RSA affects productivity by influencing the capacity of various plant functions such as symbiotic nitrogen fixation, nutrient uptake, water use efficiency [15], resistance to frost heaving [16], winterhardiness [17], and some pest and pathogen resistance [18]. Molecular and genetic breeding strategies have shown that altering the RSA of a plant can have an extraordinary positive impact on plant productivity [19, 20]. Modeling techniques in maize using historical yield data concluded that changes in root architecture were a primary driver of the nearly eightfold increase in maize grain yield in the United States since the 1930s [21]. Additionally, altering root architecture by developing maize lines with increased root depth, fewer crown roots and a decreased number of lateral root branches increases plant nitrogen acquisition, above ground growth and ultimately, yield [13]. Similar results were also observed in common bean, *Phaseolus vulgaris* [22]. In addition, increased phosphorous (P) uptake was also achieved by altering RSA. Plants limited in P acquisition tend to be limited in topsoil foraging where P, a relatively immobile soil nutrient, is known to accumulate. Therefore, alterations in RSA that produce an increase in lateral root density for topsoil foraging and reduce the growth of deeper roots, result in an increase in growth and yield as has been shown in common bean [13] and maize [23].

Modern alfalfa genotypes adapted to the upper Midwest released after 1980 have mostly a tap rooted phenotype, with little variation in the number of lateral or fibrous roots. A study on the inheritance of root morphological traits found that lateral root number, position of lateral roots, and number of fibrous roots were highly heritable traits and least affected by the environment [24]. Two cycles of divergent selection for root morphological traits with mature field grown plants were used to develop populations that were enriched for branch rooted or tap rooted plants compared to four unselected parental populations [25–27]. Moreover, the selected RSA remained stable over two growing seasons in locations differing in soil texture and fertility [18]. Forage yield was measured over 2 years in two locations for the selected and unselected populations from the four germplasm sources. For all sources, the populations selected for more fibrous and more lateral roots had greater yields than those selected for no or few fibrous or lateral roots [27]. These experiments demonstrated that changes to RSA in alfalfa are heritable, stable, and capable of increasing forage yield.

Unfortunately, field selection for root traits in alfalfa is slow, requiring a minimum of 20–22 weeks for one cycle of selection [26]. Additionally, due to the highly heterozygous and outcrossing nature of alfalfa, several cycles of selection for a specific trait are needed to develop populations with a high frequency of the desired trait. Phenotyping RSA traits in field grown mature plants is notoriously difficult due to their complex morphology, the opacity of soil, and variation in capacity for excavating a consistent root volume, among other challenges [28]. In addition, traditional manual measurement methods are constrained by the complexity and number of traits that can be feasibly measured. However, newer image-based root phenotyping methods have enabled high-throughput and accurate measurements for an effectively limitless number of traits. Using seedlings grown under controlled environments to measure RSA parameters was successful in a number of crops such as common bean [22], wheat [29], and maize [30, 31]. Using such a system in a breeding program can decrease the breeding cycles and more accurately identify the traits of interest. Studies using maize [32, 33] and common bean [22] showed high similarities between RSA at the seedling stage relative to the mature stage. These results demonstrate the high degree of congruency of RSA between the seedling and the mature plant. Such correlations support the use of seedlings grown in controlled environments as a phenotyping tool to accelerate a breeding program.

**Fig. 1:**
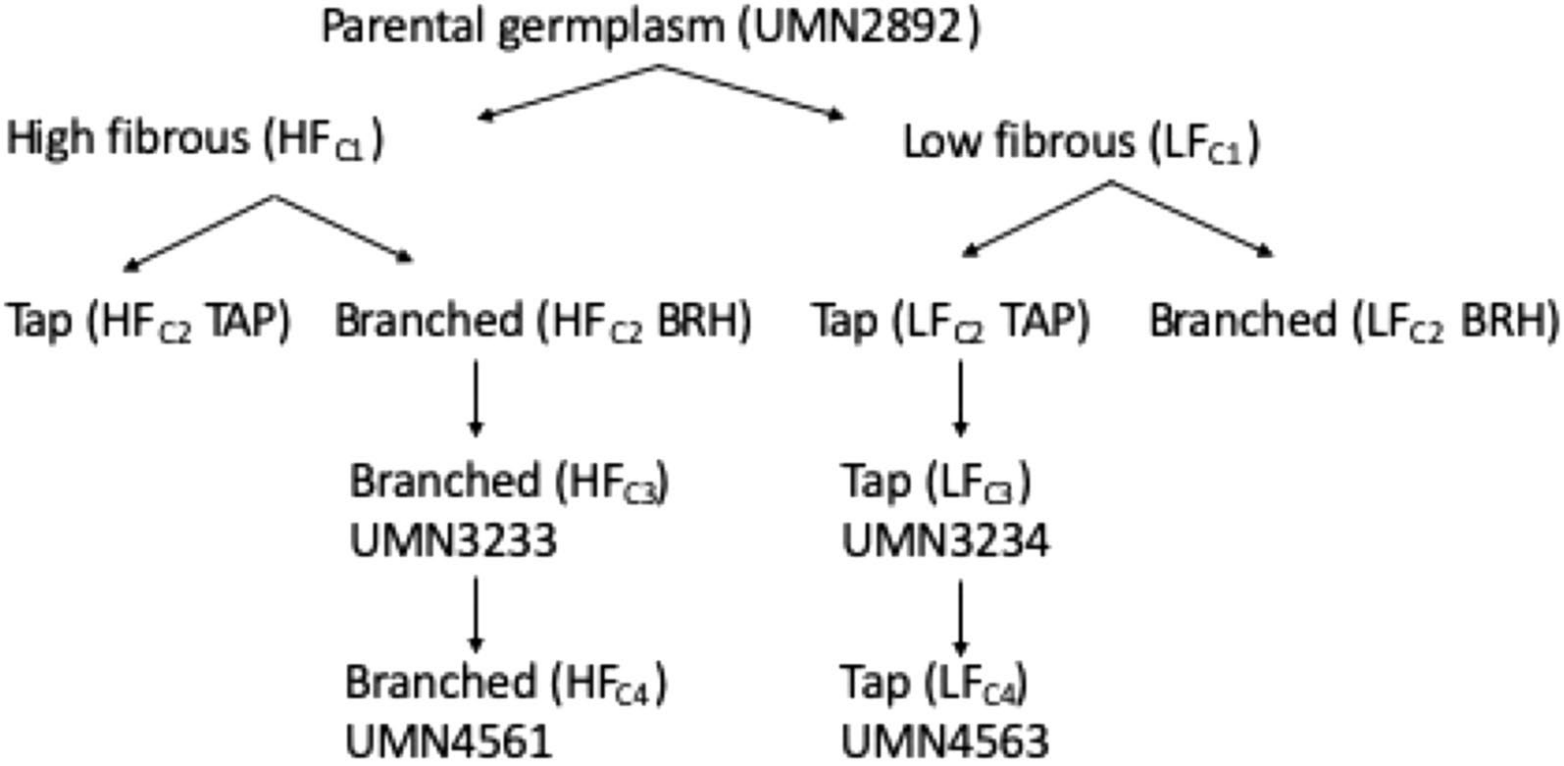
Selection for root system architecture in alfalfa. The cycle 1 (C_1_), cycle 2 (C_2_), and cycle 3 (C_3_) populations were developed by manual phenotyping for fibrous, branched, and tap roots of 22-week-old plants grown in the field. The cycle 4 (C_4_) populations were developed by selecting plants based on root traits of 14-day-old plants

This study utilized unique alfalfa populations developed from three cycles of recurrent selection under field conditions for either strongly tap rooted plants with low numbers of branched roots or highly branch rooted plants (Fig. 1). The objectives of this study were to: (1) identify measurable root traits that distinguishes the branch rooted from the tap rooted alfalfa genotypes at an early stage in development; (2) estimate the heritability of the root traits; (3) determine the correlation between root phenotypes observed at the seedling stage grown under controlled conditions to those observed in mature plants grown in the field; and (4) estimate the genetic gain achieved from the third to the fourth cycle of selection using plants selected as seedlings as the breeding stocks.

## Results

### Seedling root phenotypes from controlled growth conditions

Root phenotypes were measured from four independent experiments conducted under controlled growth conditions. Data were analyzed using analysis of variance (ANOVA) to determine if results differed between the four experiments. The results from the ANOVA showed no significant differences among the four independent experiments since the *P*-value for time equaled 0.09 (Table 1). Additionally, the *P*-value for the interaction between genotype and time was also 0.09, indicating that root phenotype was not affected by the time of the experiments. Therefore, data from the four independent experiments were combined to further examine phenotypic differences between the branch rooted and tap rooted alfalfa populations at 14 days after planting.

**Table 1:** ANOVA summary table for four independent experiments evaluating root phenotypes of 14-day-old alfalfa seedlings grown under controlled conditions

Model term	Degrees of freedom	*P*-value for tertiary root length	*P*-value for number of tertiary roots
(Intercept)	1	0.00076	0.00017
Time	3	0.24736	0.09020
Genotype	1	0.00077	0.00017
Time × genotype	3	0.24722	0.09014

**Fig. 2:**
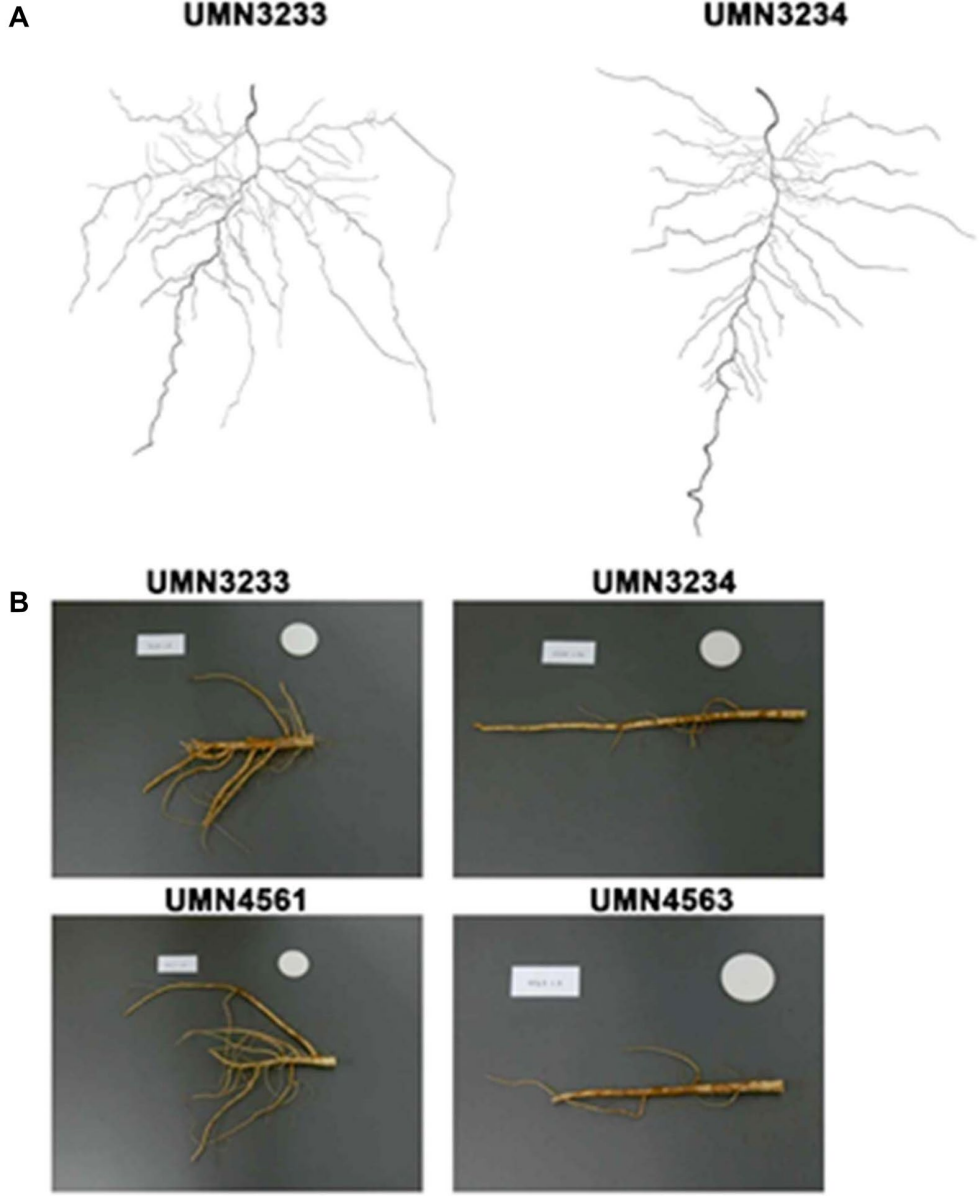
Root phenotypes of the four selected alfalfa populations. **A** Scanned images of 14-day-old alfalfa seedling roots grown under growth chamber conditions. **B** Digital photo images of roots obtained from 20-week-old field grown plants. The images are representative of progeny from the third (UMN3222 and UMN3234) and the fourth (UMN4561 and UMN4563) cycles of divergent selection. (Scale: white circle diameter = 4 cm)

**Fig. 3:**
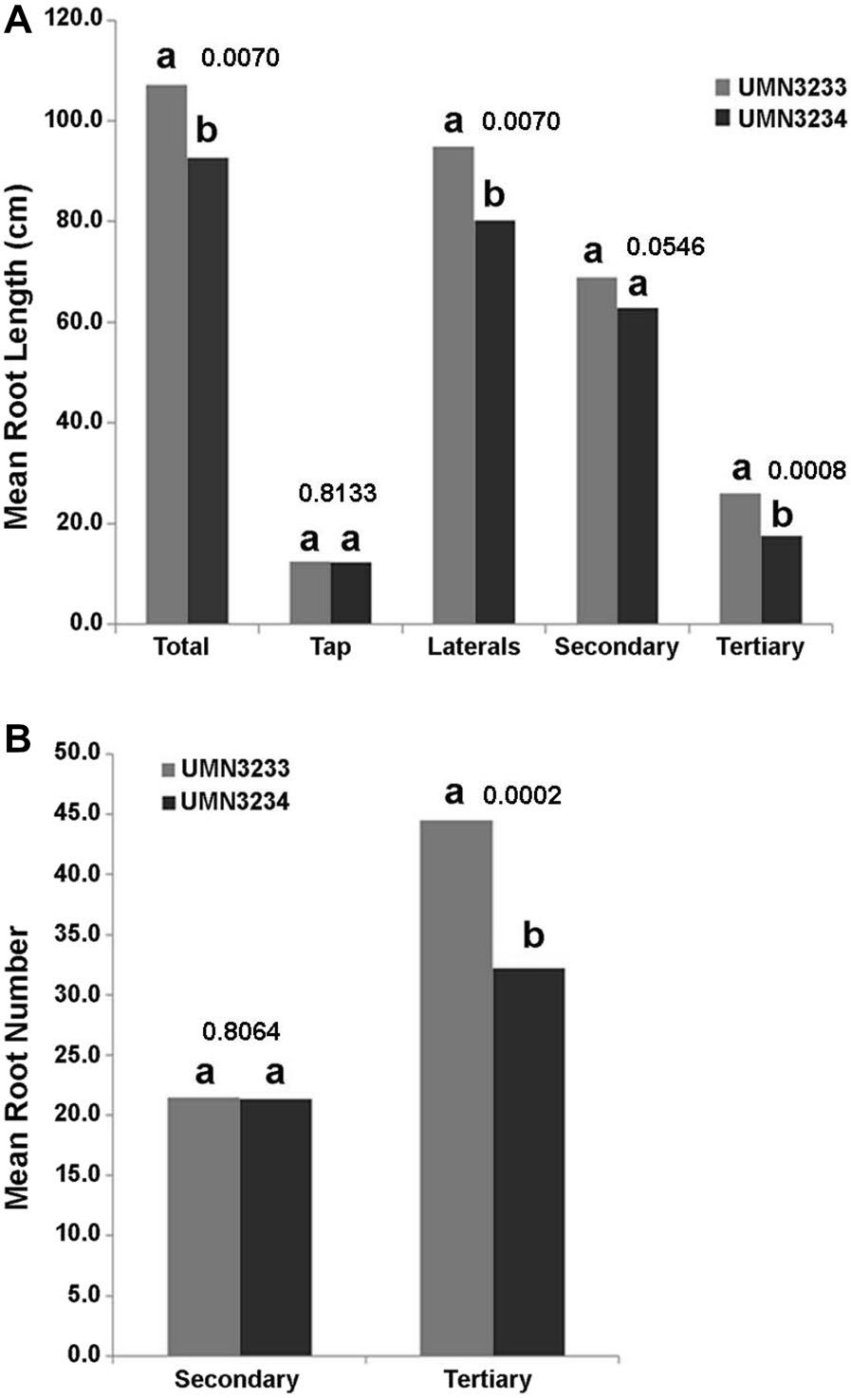
Mean root lengths (**A**) and mean root number (**B**) between alfalfa population UMN3233 (branch rooted) and UMN 3234 (tap rooted). Different letters above bars indicate significant differences by Students *t*-test (a = 0.05). Numeric values represent *P*-values. Total = total root length of all roots; Tap = Primary root; Laterals = All lateral roots; Secondary = all secondary roots; Tertiary = all tertiary roots

After 14 days of growth under controlled conditions, the seedlings of the cycle 3 populations, branch rooted (UMN3233) and tap rooted (UMN3234), had developed root systems comprised of a primary root (tap root), secondary roots (lateral roots), and the beginnings of tertiary roots, arising from positions on the secondary roots (Fig. 2A). Several measurable root parameters identified traits that significantly differentiated these two populations at this early growth stage. The most significant parameter was the difference in the number of tertiary roots. Plants from the branch rooted population (UMN3233) had a significantly greater number of tertiary roots (*P* = 0.0002) and significantly longer tertiary roots (*P* = 0.0008) relative to plants from the tap rooted population (UMN3234) (Fig. 3A and B). There were no detectible differences (*P* = 0.8064, Fig. 3B) in total number of secondary roots between the two populations, indicating that both populations produced similar numbers of secondary roots along the primary root at this early stage of development. It is worth noting that in plants from the branch rooted population (UMN3233), 51% of the secondary roots developed tertiary roots compared to only 44% in the tap rooted population (UMN3244).

The two populations showed no significant differences when total secondary root length was evaluated (*P* = 0.0546; Fig. 3A). However, a more in-depth analysis of a subgroup of secondary roots revealed secondary root length differences between these two populations. When secondary root lengths were compared for roots exceeding 2.5 cm, differences between the two populations became evident (Fig. 3A). The branch rooted population had significantly more secondary roots greater than 2.5 cm in length relative the tap rooted population (*P* = 0.00066; Fig. 3A).

Taken together, these results indicate that the major phenotypic differences in RSA for the branched and tap rooted alfalfa populations occurs during secondary root development and can be correlated to the growth and number of tertiary roots emerging along the secondary roots. However, the presence of the subset of secondary roots > 2.5 cm in length in the branched population could suggest differences may possibly be occurring earlier in development.

### Machine learning as a predictive model to identify seedling secondary root lengths that differentiate the two alfalfa populations

**Fig. 4:**
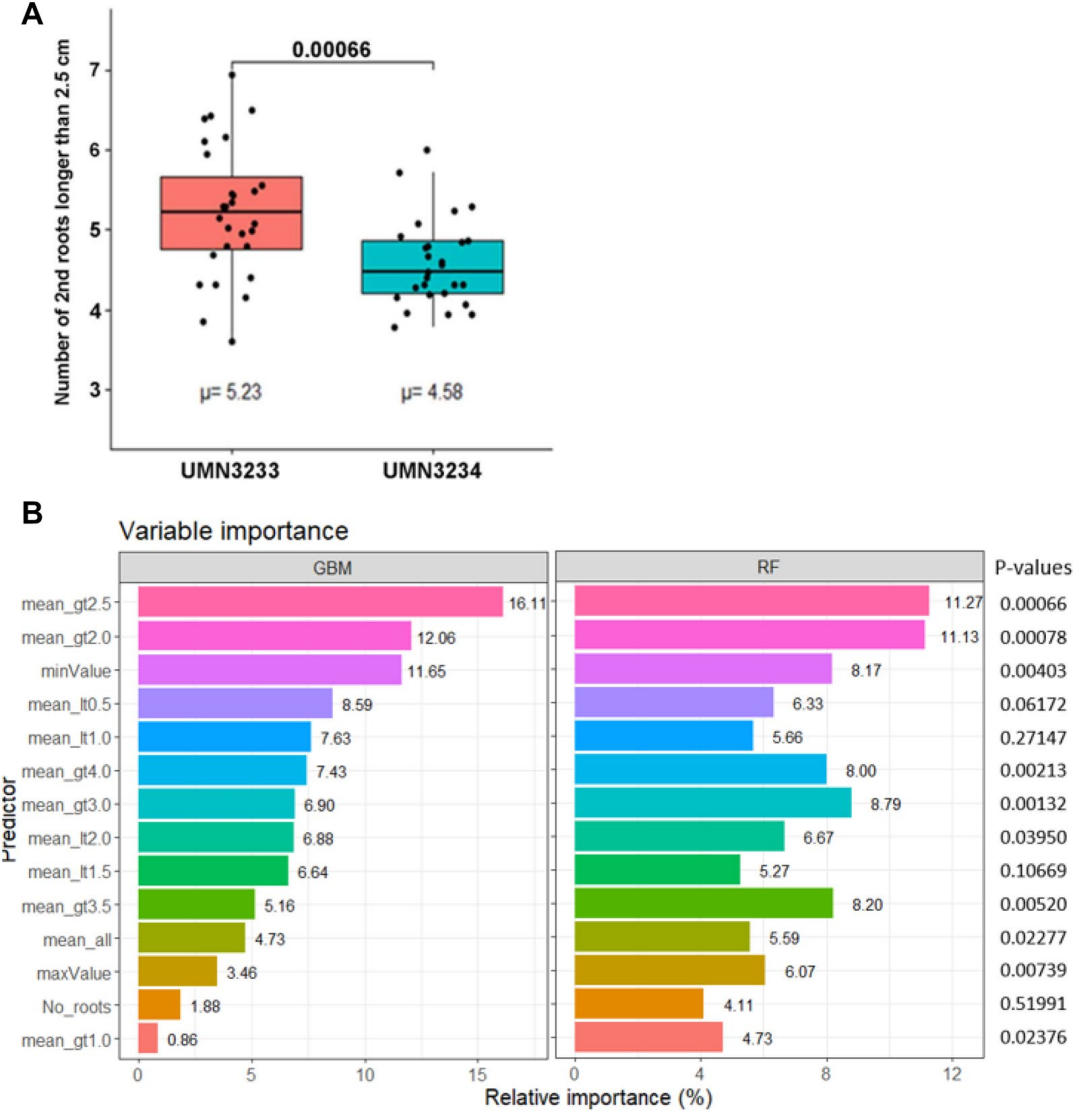
Categories of secondary root lengths that differentiate branched (UMN3233) from tap rooted (UMN3234) alfalfa populations at the seedling stage (14 days after planting). **A** Box plot showing the distributional pattern for secondary root lengths that exceed 2.5 cm. Numeric values above the plot represents the *P*-value. µ = mean number of secondary roots exceeding 2.5 cm. **B** Machine learning algorithms, GBM and RF, displaying the percent relative importance distribution for secondary root lengths exceeding specific root length values. *P*-values for each category from the t-test indicate the level of significance for each measurement between UMN3233 and UMN3234. mean_gt2.5 = mean root length > 2.5 cm; mean_gt2.0 = mean root length > 2.0 cm; minValue = minimal root length; mean_lt0.5 = mean root length < 0.5 cm; mean_lt1.0 = mean root length < 1.0 cm; mean_gt4.0 = mean root length > 4.0 cm; mean_gt3.0 = mean root length > 3.0 cm; mean_lt2.0 = mean root length < 2.0 cm; mean_lt1.5 = mean root length < 1.5 cm; mean_gt3.5 = mean root length > 3.5 cm; mean_all = mean total root length; maxValue = mean maximum root length; No_root = number of secondary roots; mean_gt1.0 = mean root length > 1.0 cm

Two different machine learning decision tree algorithms were tested, Random Forest (RF) and Gradient Boosting Machine (GBM) to create a predictive model to confirm the subset of secondary root lengths that can differentiate 14-day-old seedlings belonging to either the branched or tap rooted alfalfa populations initially identified using the statistical t-test analysis. These decision tree-based machine learning methods were applied due to their high accuracy, low overfitting, fast computation, and easy implementation. The results from both machine learning algorithms align with the results from the t-test showing the best trait to differentiate alfalfa seedlings belonging to either the branch or tap rooted alfalfa populations are secondary roots that exceed 2.5 cm in length (Fig. 4A). The GBM decision tree algorithm found that the mean number of secondary roots longer than 2.5 cm (mean_gt2.5) produced a relative importance of 16.11% (Fig. 4B). This trait was also identified among the top traits by the RF algorithm, with a relative importance of 11.27%. These results from both machine learning algorithms validate the results from the t-test analysis (*P* value = 0.00066), confirming that the subset of secondary roots > 2.5 cm in length were significantly different between the two alfalfa populations at the seedling stage. Such a strong correlation between these two independent tests corroborates the approach of using alfalfa seedlings, as early as 14 days after planting for selection of root traits.

Several differences were observed in the results from variable analysis between GBM and RF. The percent relative importance of the traits resulting from GBM are much larger than from RF. The largest and smallest relative importance from GBM were 16.11% for mean_gt2.5 and 0.86% for mean_gt1.0 (mean secondary root greater than 1.0 cm) with the difference being 15.25. In contrast, the largest and smallest relative importance of traits with RF were 11.27% for the mean_gt2.5 and 4.11% for the No_roots (number of secondary roots) with a difference of 7.16%. The mean_gt3.0 (mean secondary root greater than 3.0 cm) from RF is the third most important variable with *P* = 0.00132, whereas it was ranked only as number seven by GBM.

### Heritability of alfalfa seedling root traits 14 days after planting

An analysis of the estimate of heritability for the seven root parameters measured in seedlings of the two alfalfa populations that display either a branched or tap rooted phenotype showed that the highest heritability estimate is associated with the number and length of developing tertiary roots, 0.7885 and 0.7415, respectively (Table 2). In contrast, the heritability values for tap root length and secondary root number approached zero (Table 2). Intermediate heritability estimates were found for total root length (0.5938), total lateral root length (0.5987), and total secondary root length (0.3250).

**Table 2:** Heritability of alfalfa seedling root traits grown under controlled conditions and evaluated at 14 days after planting

Root parameter	Heritability
Total root length	0.5938
Tap root length	0.0001
Total lateral root length	0.5987
Secondary root length	0.3250
Tertiary root length	0.7415
Secondary root number	0.0001
Tertiary root number	0.7885

These results indicate that the growth and development of tertiary roots at this early stage in plant development are highly linked to genetically inheritable traits and are favorable candidates to be used in future breeding experiments. In contrast, the traits with the lowest values, tap root length and secondary root number, represent traits most likely affected by environmental factors.

### Principle component analysis (PCA) and its significance to root trait inheritance/stability in alfalfa seedlings

**Fig. 5:**
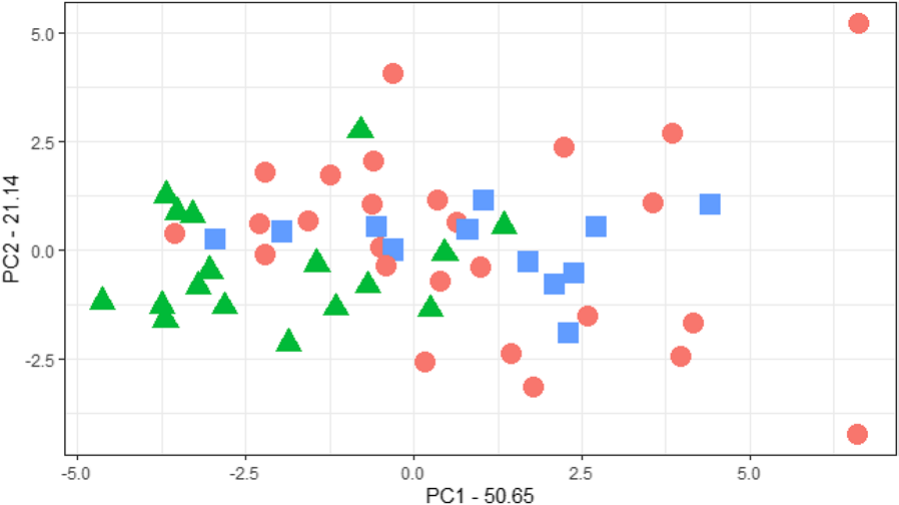
Principal component analysis (PCA) of alfalfa root phenotypes from UMN3233 and UMN 3234 seedlings at 14 days after planting. Root system architecture was categorized as branch rooted (red circle), tap rooted (green triangle), or an intermediate phenotype (blue square). The top three principal components explained 50.65%, 21.14%, and 7.28% of the variance in seedling root architecture

Roots from alfalfa seedlings (UMN3233 and UMN 3234) grown under controlled conditions were categorized by visual phenotyping of imaged roots (Fig. 2A) as branch rooted, tap rooted, or an intermediate phenotype. This visual phenotypic data was evaluated using PCA. Results of PCA show relatively close clustering of tap rooted plants and intermediate phenotypes while branch rooted plants are less tightly grouped (Fig. 5). Additionally, overlap of the three phenotypes suggests segregation of these traits within these plant lines is still occurring. The first three components account for 50.65%, 21.14%, and 7.28% of the phenotypic variation, respectively.

### Phenotyping and genetic gain estimation of field grown alfalfa populations at maturity

**Fig. 6:**
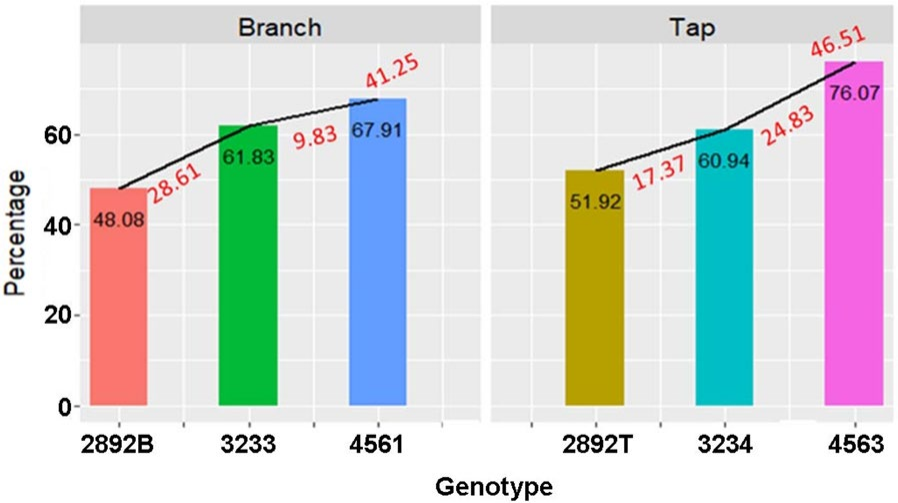
Genetic gain for selected alfalfa populations with either branched or tap rooted populations. The numbers in black, within the bars, correspond to the percentages of the root type for the specific population. The numbers in red, below the black line, are genetic gain values between the selection cycles. The number in red, above the black line is the genetic gain from all three selected populations combined

Plants selected at the 14-day-old seedling stage for branched or tap roots were used in crossing blocks to develop cycle 4 populations. These cycle 4 populations, the parental population (UMN2892), and populations derived from the third cycles of selection were grown from seed under field conditions and scored for root traits after 20 weeks of growth. Plants from the fourth cycle of selection, UMN4561 and UMN4563, had significantly higher percentages of plants that exhibited the branch or tap rooted phenotype relative to plants from the third selection cycle (P < 0.05; Fig. 6). The percentage of plants exhibiting a branched rooted phenotype from the fourth cycle (UMN4561) was 68% relative to 62% for plants from the third cycle (UMN3233). Likewise, the percentage of plants exhibiting a tap rooted phenotype from the fourth cycle (UMN4563) was 76% compared to 61% for plants from the third cycle (UMN3234). Genetic gain of the branch rooted type was greatest between the parental population and the population from the third cycle while genetic gain of the tap rooted type was greatest from the third to fourth cycle of selection. Overall, genetic gain from the parental population to the fourth cycle was 41% and 47% for the branched and tap rooted phenotypes, respectively (Fig. 6). The average genetic gain was approximately 10% at each cycle. These results show that the use of seedlings for selection was effective for increasing the desired traits in each population.

## Discussion

The results of these experiments showed accurate root type discrimination in alfalfa at 14 days after planting, reducing the time from the conventional phenotypic selection by 20 weeks. Additionally, seedling selection increased the proportion of the tap rooted trait to a greater extent than adult plant phenotyping. With this rapid selection method, it is possible to complete two cycles of selection in a single year, although field phenotyping for other agronomically important traits would still be required during the breeding and cultivar development process.

In the expanding area of plant phenomics, the development of tools to evaluate roots and other below ground structures has lagged behind the development of tools for evaluating forage and above ground traits. Challenges to root evaluation include the lack of correlation of RSA in controlled conditions or artificial growth medium compared to field conditions. For example, Lamb et al. [26] compared taproot diameter, lateral root number, and fibrous root mass in four alfalfa germplasms under field and greenhouse conditions. The only trait that could reliably be assessed under greenhouse conditions was taproot diameter. For all other traits assessed, there was no significant correlation between greenhouse grown plants and field-grown plants. Similarly, no correlation of traits between field grown plants and plants transplanted from the greenhouse to the field was identified. Additionally, transplants had a higher frequency of determinate taproots than plants directly seeded into the field, indicating that transplanting caused root injury and loss of apical dominance in the injured root. In field grown plants, the transition from juvenile to adult plant root traits occurred at 14–17 weeks after seeding, and 22 weeks of growth under field conditions was recommended as the minimal age for characterizing taproot diameter and lateral root number.

In addition to the time required, phenotyping plant root systems in the field is also costly, time-consuming, and typically involves destructive sampling. Excavation of plants and manual phenotyping can be complicated by use of different rating scales, is limited in the number of traits that can be evaluated, and is subject to human error, although digital image analysis can simplify collection of data and increase the number of traits evaluated [34]. For some plant species, RSA and root morphology have a high degree of plasticity [35], affected by soil structure as well as nutrient and water availability [36–38]. Plant growth promoting bacteria [39] and rhizobia [40] have also been found to affect RSA. Nodulation of alfalfa with the nitrogen fixing bacterium *Sinorhizobium meliloti* alters root morphology [41], although it is still an open question on whether root architecture affects nodulation. Selection of alfalfa for tap rooted and branch rooted traits appears to be stable across environments and soil types [18].

Some noninvasive methods for phenotyping roots in the field such as ground penetrating radar are being developed that can accelerate breeding cycles [42], although they may lack the high throughput needed for phenotyping large populations, and their utility for phenotyping alfalfa that has both thick and fine roots is untested. Goins and Russelle [43] used rhizotrons to evaluate root traits in tap rooted and branch rooted alfalfa over a field season. Although the branch rooted types had 29% more fine roots at 20 cm than the tap rooted types, no other root trait measured by this method could distinguish the two root types. Almost all root phenotyping has been done using annual plant species. The development of perennial root systems over multiple years has yet to be fully explored.

The results of this study demonstrated that using various measurable root parameters obtained from alfalfa seedlings, 14 days after planting and grown under controlled growth chamber conditions, is a viable option for selecting alfalfa individuals with unique root phenotypes. Also, using plants selected as seedlings in a crossing block resulted in a field grown populations that had an approximate 10% increase in the number of individuals with the specific root phenotype of interest relative to the previous selection cycle. This demonstrated that the root trait characteristics in alfalfa are stable between growth chamber and field grown environments and are highly heritable.

The advantage of using seedlings as an indicator of a mature plant phenotype has been reported in both monocots and dicots [22, 32, 44, 45]. Such studies have shown a high correlation between root architecture of young and mature plants, grown under both controlled and field environments and the association between RSA and plant yield. Strock et al. [22] phenotyped a large and diverse population of common bean and compared the RSA of seedlings grown in various diverse field environments. They found correlations to be exceedingly high, in some cases approaching 0.6, between the RSA of plants at both stages of development regardless of their growth environment. Likewise, comparisons of RSA in diverse maize inbred lines under controlled and field conditions confirmed the positive correlations in RSA between seedlings and adult plants [32, 46].

Johnson et al. [47] observed wide variation in alfalfa root morphological traits within plant introductions and North American varieties suggesting that selection for specific root modification is feasible. Nonetheless, modern alfalfa cultivars adapted to the upper Midwest released after 1980 are mostly tap rooted with little variation in the number of lateral roots or fibrous root mass. Barnes et al. [48] found that lateral root number, position of lateral roots, and number of fibrous roots were traits least affected by the environment and Johnson et al. [24] found high heritability for these traits. Even though progress was made in trait selection and the two diverse genotypes with either a branched (UMN4561) or taproot (UMN4563) phenotype were successfully selected through four divergent selection cycles, the two genotypes continue to segregate in their root phenotype. The percentage of plants from the fourth cycle of selection that displayed a branched root phenotype (UMN4561) was 68%. Similarly, for the fourth cycle of selection for a taproots (UMN4563), 76% of the plants displayed the phenotype. These results indicate the challenges in breeding for RSA with low phenotype-based selection rates. Identification of QTLs associated with root type or genomics-based marker-assisted selection is needed to increase accuracy in selection for root traits. Mapping of QTLs for RSA and root traits has been successful in other crops. Cai et al. [49] identified QTLs in maize for RSA using both young seedlings and mature plants grown in the field. Mace et al. [45] mapped sorghum root angle QTLs under controlled conditions associated with drought tolerance. Sanchez et al. [31] used 14-day-old maize seedlings, grown under controlled conditions, in a GWAS study that identified markers associated with early root development. Additionally, such results point to a strong interaction between robust root architecture traits, plant yield, and root development, thus confirming a genetic link between these traits [50]. Collectively, these studies support the efficacy of using seedlings in traditional breeding programs to accelerate the breeding process as well as studies to identify candidate genes underpinning the trait(s) of interest. The selected alfalfa populations developed in this research will be valuable for future work to identify and validate associated QTLs and causative genes.

In this study, secondary root length greater than 2.5 cm was the major phenotypic difference between branch rooted and tap rooted plants and this trait was confirmed by machine learning to be the most informative to distinguish root types. Such measurable differences between these two root types suggest distinct genetic and/or hormonal differences. Root development and growth have been studied extensively in Arabidopsis [51]. Lateral or secondary root development mimics the organogenesis of primary root development, but little is known about the regulation of tertiary root formation. In lateral root formation, auxin plays a key role in integrating external and internal signals with root development mainly controlled by several auxin-regulated transcription factors. Perotti et al. [52] found that secondary and tertiary roots in Arabidopsis exhibit different gene expression patterns and developmental programs. The transcription factor AtHB23 is expressed throughout the tertiary root primordium and appears to play a key role in regulating tertiary root formation. Gene expression studies from different root segments and multiple developmental time-points utilizing the branch rooted and tap rooted alfalfa lines could increase understanding of tertiary root initiation and development.

Breeding for specific root traits has a number of advantages for improving alfalfa cultivars. Herbage yield and stand persistence of alfalfa may be enhanced by selecting for specific root traits. The alfalfa root system, particularly the taproot, acts as a storage organ to supply carbon for regrowth after harvest of herbage and for spring regrowth. The perennial nature of alfalfa is due in part to the indeterminant meristems in roots [53]; however, the contribution of root traits such as taproot diameter or root dry matter has not been explored to increase winter survival and persistence in alfalfa. A deep tap root also increases potential access to water resources to improve drought tolerance [54] and reduce competition from shallow rooted plants such as forage grasses that may be interplanted with alfalfa. Branched roots increase nutrient acquisition [55], nitrogen fixation [56], and wet soil tolerance [57]. The materials developed in this study will be used in future research to investigate the advantages of branched or tap rooted plants for enhancing agronomic traits in alfalfa.

## Conclusions

In this study, developmental differences between branch rooted and tap rooted plants were identified based on seedling phenotyping. The use of alfalfa seedlings to select for divergent root traits is a viable strategy to alter RSA at plant maturity and accelerate the breeding cycles driving towards specific root phenes. Future selections for these root traits that include a marker-assisted strategy would further streamline the breeding process. This strategy would also help elucidate and correlate causative genes for the trait of interest and further explore the developmental mechanisms associated with these traits.

## Methods

### Plant materials with divergence root system architecture

Two experimental alfalfa populations (UMN3233, UMN3234) originating from the breeding program as described by Lamb et al. [25] were used in this study. These populations were developed from MWNC (UMN2892), a composite of eight dormant experimental populations selected for resistance to Phytophthora root rot (*Phytophthora medicaginis*), Aphanomyces root rot (*Aphanomyces euteiches*), and the root-lesion nematode (*Pratylenchus penetrans*). The parental germplasm underwent a cycle of divergent selection for amount of fibrous root mass, creating populations low in fibrous root mass (LF) and high in fibrous root mass (HF), followed by a cycle of selection for tap roots (TAP) and lateral branch roots (BRH), creating low fibrous tap rooted (LF_C2_TAP) and highly fibrous branch rooted (HF_C2_BRH) populations. Lastly, these two populations underwent a third cycle of selection for fibrous root mass to create UMN3233 (from 265 parental plants) with high fibrous branched root mass and UMN3234 (from 212 parental plants) with low fibrous taproots (Fig. 1). In each cycle of selection, the selected plants were randomly intermated within the phenotypic group and the resulting progeny evaluated for root traits. Those plants with the desired phenotypes within each group were used as parents for the next cycle of intermating. All plants were selected in field plots after 22 weeks of growth from seeds and scored visually for lateral root number and fibrous root mass [25].

### Seed germination and plant growth under controlled growth chamber conditions

Seeds from UMN3233 and UMN3234 were sandpaper scarified and germinated at room temperature on moist filter paper for 24 to 48 h. Individual germinated seeds were planted in 7.5 × 35 cm plastic cone-tainers (Stuewe & Sons, Tangent, OR) containing a 1:1 (v/v) mixture of pasteurized sand and Turface MVP (Profile Products, Buffalo Grove, IL). In preliminary experiments, this mixture of sand and Turface was found to reproducibly generate plants with the expected root phenotypes that could be removed from the growth medium without damage to roots. Seedlings were placed in a growth chamber with a 16-h photoperiod at 24 °C and light intensity of 380 µmol m^−2^ s^−1^. Cone-tainers were covered with clear plastic for 5 days to promote plant establishment. Plants received 70 ml of 0.5X Hoagland’s nutrient solution, pH 7 with 100 ppm NO_3_ every other day [58]. On alternate days 70 ml of deionized water was applied. After 14 days, the plants were gently removed from the cone-tainers and roots were washed to remove any attached soil particles. Roots were maintained between moist paper towels prior to phenotyping as described below.

Four growth chamber experiments were conducted with each experiment treated as a repeat block. Plants from the two populations, UMN3233 and UMN3234, were randomly assigned to cone-tainers within each block. A total of 88 root systems from individual plants were analyzed in the study. Three experiments used a total of 16 plants, eight plants of UMN3233 and eight plants of UMN3234. In the fourth experiment, 20 plants of each population were randomly assigned to cone-tainers.

In order to retain plants to be used for a fourth cycle of intermating, shoots from selected individuals displaying the strongest branched or tap rooted phenotypes were excised and placed in moist medium grade vermiculite in the growth chamber to initiate adventitious root production. Once rooted, the plants were transferred to pots containing a 3:1 (v/v) mixture of pasteurized soil: sand and grown in the greenhouse until they produced flowers. Plants were randomly intermated by hand pollination within the two selected groups and seed collected from the female parents. Equal amounts of seed from 10 female parents of the branch rooted group were combined to form UMN4561 (HF_C4_BRH) and equal amounts of seed from 12 female parents of the tap rooted group were combined to form UMN4563 (LF_C4_TAP) (Fig. 1).

### Root phenotyping

Intact root systems were digitized with a STD4800 flatbed scanner operated by the WinRhizo software (Regent Instruments Inc., Canada). The image acquisition parameters for WinRhizo, using the indicated scanner, were as follows: The roots were scanned in gray scale, with a scanner resolution setting of 400 dpi [59]. The Internal Regent Positioning System was activated and linked to a 20 × 25 cm tray size. Root classifications were identified and measured using the Developmental Analysis tool in the software. Lengths for each scanned root system were calculated from the following root classification types: tap root (primary root), secondary roots, and tertiary roots. The secondary root is defined as a branch of a primary root (or tap root) and a tertiary root is defined as a branch of a secondary root. Total root length, length for each individual root and for each root classification was obtained. Tap root length was measured from the severed point below the cotyledonary node to the distal root tip.

### Field growth conditions

The root architecture of the parental germplasm and progeny from the third and fourth cycles of selection of plants, UMN3233, UMN3234, UMN4561, and UMN4563, were evaluated under field conditions. The four experimental populations were individually hand seeded into 1.4 m × 0.9 m plots with 28 plants per plot. The plants were equally spaced within the plot using a 13 cm × 13 cm grid. All grid positions were seeded with two to four seeds and thinned to one plant at 21 days after seeding. Each plot was surrounded by a border row of the alfalfa cultivar Agate. Six replicated plots per population were randomly spaced within the field. Planting was done on 1 June 2016 at the University of Minnesota St. Paul Experiment Station (Waukegan fine-silty loam: sandy-skeletal, mixed, superactive, mesic Typic Hapludoll). The plant root system was excavated 20 weeks after planting by digging individual plants to a depth of 12 to 18 inches on 12 October 2016. Foliage was removed 4 cm above the crown. Roots were washed to remove soil and stored at 4 °C. Root systems were photographed with a 4 cm diameter size marker using a digital camera and root phenotypes were categorized based on the images. The branch root phenotype was categorized as producing four to six lateral roots along the tap root at 1–2 cm intervals. The taproot phenotype was categorized as having less than four lateral roots emerging from the tap root that were spaced 3–4 cm apart. The total number of roots from individual plants evaluated for each population ranged from 94 to 129.

### Statistical analysis

Data from the four growth chamber experiments were pooled to compare the two alfalfa populations (UMN3233 vs. UMN3234) using the PROC TTEST in SAS (version 9.3). Data from field experiments were analyzed using the MIXED procedure of SAS. Alfalfa population and root phenotypes were considered as fixed effects, and replicates and interactions with replicates were considered as random effects. Residuals were checked for normality and homogeneity of variance using UNIVARIATE procedure of SAS and scatterplots of residuals vs. predicted values [60]. Data were logarithm base 10-transformed prior to analysis to meet these assumptions, as required. Mean difference comparisons were conducted via model selection of “t.test” from the R package “ggpubr” [61] and SAS software. Principal component analyses were conducted with the “prcomp” function from base R package “stats” using the R Studio Version 1.3.1093 [62]. The 3D plot was visualized via the R package “pca3d” [63].

Broad sense mean based heritability (H^2^) [64] is given as:

$${\text{H}}^{2} = {\text{Vg}}/{\text{Vp}} = {\text{Vg}}/\left( {{\text{Vg}} + {\text{Ve}}//{\text{r}}} \right) = {\text{Vg}}/\left( {{\text{VA}} + {\text{VD}} + {\text{VI}} + {\text{Ve}}//{\text{r}}} \right)$$

where Vg stands for genotypic variance, Vp for phenotypic variance, VA for the variance of the additive effects, VD for the variance of dominance effects, VI for the variance of epistasis (the gene interaction), Ve is the variance of the residuals, and r for the number of replicates for each measurement.

Root traits were identified by machine learning decision tree algorithms, Random Forest (RF) and Gradient Boosting Machines (GBM). The GBM analysis was implemented via R package “gbm” with the number of decision tree “n.tree” = 500 and number of cross-validation “cv.folds” = 10 [65]. The RF algorithm was employed to cross verify the traits selected from the GBM via the R package “RandomForest” with number of the decision tree “ntree” = 1000 [66].

## Data Availability

The datasets used and/or analyzed during the current study are available from the corresponding author on reasonable request.
